# Assessment of farmers’ knowledge and perceptions of coffee yield reduction due to weeds and their management in Ethiopia

**DOI:** 10.1016/j.heliyon.2023.e19183

**Published:** 2023-08-18

**Authors:** Abera Daba, Mekuria Tadesse, Minilik Tsega, Gezahegn Berecha

**Affiliations:** a Department of Horticulture and Plant Sciences, Jimma University, Jimma, Ethiopia; b Department of Horticulture, Wollega University, Nekemte, Ethiopia; c Ethiopian Institute of Agricultural Research, Addis Ababa, Ethiopia; d Department of Horticulture, Wolkite University, Wolkite, Ethiopia

**Keywords:** Arabica coffee production culture, Yield loss, Weeds, Weed management, Canonical correlation analysis

## Abstract

The home of *Coffea arabica* is in Ethiopia, where it has high genetic diversity and suitable growing conditions; unfortunately, the national average yields of coffee remain low due to no technical advancements and diverse, complex biotic and abiotic constraints. Hence, this study was conducted in eight major coffee-growing zones of Ethiopia to assess farmers' knowledge and perceptions of coffee yield reduction due to weeds and the farmers' weed control practices. A purposive and random sampling technique was used to generate primary data from coffee growers (*N* = 320) using a semi-structured questionnaire. Quantitative data were analyzed using a three-stage nested design, and the dependent and independent variables data were subjected to canonical correlation analysis. This study revealed variation in coffee yield (t ha^−1^) among the assessed areas based on farmers’ knowledge of estimating coffee yield. The average yield level ha^−1^ was very low (0.37 t ha^−1^) and different among the surveyed areas. The average coffee yield gap as compared to the current national level (0.64 t ha^−1^) was observed to be 42%, and this low yield was highly correlated with weed infestation (r = 0.879) and type of weeds r = −0.528). This investigation indicated a single factor or association of different factors contributing to the low yield level of coffee in the study areas. Thus, it is concluded that predictor variables accounting for the low yield levels need to be considered when planning future strategies to attain the yield potential of *C. arabica* in Ethiopia.

## 1 Introduction

Coffee is one of the most widely traded tropical commodities in the global market [1]. The two commercially produced and traded coffee species are *Coffea arabica* and *Coffea canephora* [2]. The former is the most important cash-generating crop with significant economic value for Ethiopia, and the country ranks first in Africa and the fifth-largest producer in the world [3]. The main coffee-growing regions are in the South-west and Southeast, namely the Oromia Region and Southern Nations, Nationalities and People Region (SNNPR), with modest production in the Amhara Region and minor production in the Benishangul-Gumuz Region [4]. Coffee production covers approximately 538,000 ha of land, and 447,000 t of coffee yield was obtained in the 2019/20 main production season [5].

While Ethiopia is the home of *C. arabica* with high genetic diversity and suitable environmental conditions, the national average yields remain low, estimated at 0.64 t ha^−1^ [6]. This low yield has been attributed to poor technological advancements and diverse and complex biotic and abiotic constraints, which reduce the crop's quantity and/or quality. Furthermore, the lack of infrastructure, the absence of improved coffee variety, and the lack of extension services were major constraints on coffee production in the country [7]. The crop losses caused by weeds have exceeded the losses from any category of agricultural pests [8]. Weed growth and competition affect coffee yield and quality, particularly in organic coffee production, where synthetic herbicides are prohibited [9]. Unlike diseases or insect pests, where yield loss might be possible due to those individual pests [10], yield loss estimation due to a single weed species is practically impossible; hence, it is estimated as the collective impact by all weeds. Weeds are very prolific in multiplication, and if allowed to grow in coffee, they are very competitive for soil moisture, light, and essential nutrients [11]. The studies by Eshetu et al. [12] showed that the yield loss assessment for coffee was 60–80% in Jimma, Ethiopia. Similarly, coffee yield loss in Kenya can be over 50%, leading to a total loss if not managed [13]. These studies imply that weeding is a vital operation in coffee production.

As Kumar et al. [14] reported, crop loss assessment quantifies the impact of pests on crop yield. Quantitative information on crop loss assessment is necessary and the first step toward crop health management [15] as it provides key information for developing sustainable crop management systems [16]. In weed management strategies, a field survey can address the problem in farmers' fields and build target-oriented research programs [17]. Hence, it is evident that yield loss assessment is very important to formulate appropriate strategies to attenuate crop loss and increase farmers' income. Most of the studies conducted in the past concerning coffee yield loss assessment in Ethiopia mainly focused on yield loss due to diseases and insects with available control practices. However, coffee yield losses due to weeds and practices that farmers use to manage weeds are not well documented. Current available information is outdated and does not apply to all major coffee-growing areas in Ethiopia. Therefore, there is a need to explore the information gap about coffee production, yield loss due to weeds, and control practices used by smallholder farmers. Therefore, this study aimed to assess farmers' knowledge and perceptions of coffee yield reduction due to weeds and farmers’ weed control practices across the major coffee-growing zones of Ethiopia.

## 2 Materials and methods

### 2.1 Description of the study area

**Fig. 1:**
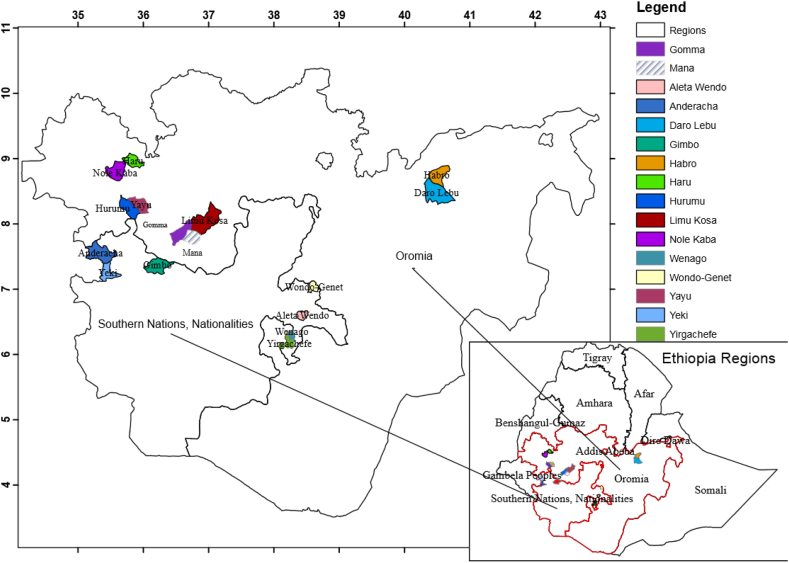
Map showing 16 coffee growing districts in 8 zones in Oromia and SNNP regions of Ethiopia.

A survey was conducted in two major coffee-growing areas of Ethiopia, namely Oromia, and SNNPR, which represent 99% of coffee production (Fig. 1; [4]). Of the top 25 coffee-producing districts, 18 are found in Oromia (of which 5 are in the Jimma zone), and the remaining 7 are in SNNP [18]. The surveyed areas ranged from 1200 to 2080 m above sea level (masl) and 7° 44′ to 8° 55′N, and 35^0^ 00′E to 40° 28′E. The mean annual temperature is within the optimum level ranging from 17.40 to 22.80 °C, while the mean annual rainfall in the study areas ranges from 921 to 1870 mm, and a summary of climatic conditions was displayed in our previous report [19].

#### 2.1.1 Sampling techniques and sample size

A survey was conducted from June to September 2018 in the selected coffee-growing zones of Ethiopia to assess farmers' knowledge and perceptions of coffee yield and coffee yield reduction due to weeds and farmers’ weed control practices. Two sampling techniques were used: in the first stage, the purposive sampling technique was used to select two regions (Oromia and SNNPR), four administrative zones from each region, and one to three districts from each zone were selected based on the production status of the crop. Accordingly, Mana, Gomma, and Limu Kosa districts from the Jimma zone; Yayu and Hurumu from the Illubabor zone; Haru and Nole Kaba from West Wollega and Abro and Daro Lebu from the West Hararghe zone were selected. Similarly, from the SNNP region, Yeki and Anderacha (Sheka zone); Gimbo (Kaffa zone); Wenago and Yirgachefe (Gedeo zone); Wondo Genet and Aleta Wendo (Sidama zone) were considered for the survey. Then by considering the size and accessibility of the areas (adjacent to the road), two to three kebeles (neighborhoods) per district were selected purposively. In the second stage, a simple random sampling technique was applied to select household farmers (target group) from each kebele (except for forest and plantation coffee systems where the farms were intentionally selected). In total, 320 coffee growers were selected to be surveyed using the method of Daniel [20], as previously described.

#### 2.1.2 Methods of data collection

A cross-sectional data collection method was carried out through surveys involving individual semi-structured questionnaire interviews based on a literature review and pre-interviews with model farmers, government offices, the manager of a large coffee estate, local administration, and several scientists working with coffee-based including development agents (DAs) in the country. The semi-structured questionnaire once drawn up, was pre-tested and administered by trained enumerators recruited from the target districts with good knowledge of the areas of the study. Before beginning the interview, farmers learned about the study's objectives and indicated their acceptance to be interviewed. Once they agreed, each coffee grower was interviewed with open-and close-ended questions to determine the dominant socio-demographics and their knowledge and perception in relation to coffee production practices, yield loss due to weeds, weed management practices they employed, and other information related to their coffee production system (See supplement S1 for survey questionnaire).

Weed infestations and the effectiveness of weed management practices were determined by walking around the coffee fields and making observations during the survey period. Weed management practices were coded on a scale of 0–3, with 0 meaning no weed management, e.g., forest coffee, 1 meaning slight weed management where farmers remove weeds once each year, 2 meaning a moderate level of weed management, that removed weeds twice a year and 3 meaning the best weed management where farmers continuously removed weeds from the coffee field. Weed infestations were coded on a scale of 0–3, with 0 meaning no weeds in the field, 1 meaning slightly infested, 2 meaning moderately infested, and 3 meaning heavily infested, as described in Muller et al. [21].

Farmers identified the most troublesome weeds by their local names. Taxonomical names and clarification were confirmed using the Floras of Ethiopia and Eritrea [22,23]. Data from the semi-structured questionnaires were supplemented by focus group discussions (FGDs), each with eight to ten members. The focus group identified strategies and techniques that coffee growers, DAs, and other expertise found useful for managing weeds in coffee fields. Respondents were asked to estimate yield level and extent of yield loss based on fresh and dried cherries and then converted to green beans. Even though there was no clear established standard for the conversion of fresh and dried cherries to clean dried beans of *C. arabica* in the country, for this particular study, a ratio of 6:3:1 (6 kg of fresh cherries makes 3 kg of dried cherries that produce 1 kg of clean dried beans) [24].

#### 2.1.3 Data analysis

The demographic and socio-ecosystem data were summarized, and descriptive data analysis was done using means, frequencies, and percentages. Data on farmers' knowledge and perceptions of coffee yield and coffee yield reduction due to weeds and farmers' weed control methods were analyzed using a three-stage nested design. Analysis of variance (ANOVA) was performed using SAS software version 9.4 (SAS Institute Inc., Cary, NC, USA). For significant parameters, means were separated using Tukey's test at a 5% significance level.

The association of coffee yield level and crop yield reduction (based on farmers’ knowledge and perceptions) with independent variables (age of coffee trees, coffee production systems (CPS), level of weed infestation, types of weeds, weed management practice, weeding frequency and types of coffee variety cultivated) was computed using canonical correlation analysis to establish their relationship using SAS software version 9.4 (SAS Institute Inc., Cary, NC, USA). Canonical correlation analysis is a multivariate method that includes regression and multivariate analysis of variance in the general linear model [25]. The method allows for simultaneously examining the relationship between several predictors and dependent variables [26,27].

When variables were strongly correlated (>0.7), only one of them in the pair was included in the model. Similarly, variables with values of tolerance above 0.10 and the smallest variance inflation factor (VIF) values (<5) were considered in the model. Wilk's multivariate Lambda (λ) significance test (*F* distribution approximation) was used to evaluate the significance of the canonical roots. For this study, the two sets of variables (X-independent) and (Y-dependent) are defined as follows.

$$X_{5x1}=\left\{ \begin{array}{c} X_{1}=Weed\;infestation\\ X_{2}=Types\;of\;weeds\\ X_{3}=Improved\;variety\\ X_{4}=CPS\\ X_{5}=Age\;of\;coffee\;trees \end{array}\right.,Y_{2x1}=\left\{ \begin{array}{c} Y_{1}=Yield\;level\\ Y_{2}=Yield\;loss \end{array}\right.$$

## 3 Results

### 3.1 Characteristics of coffee production socio-ecosystem

#### 3.1.1 Cropping sequences ecosystems managed by coffee growers

**Fig. 2:**
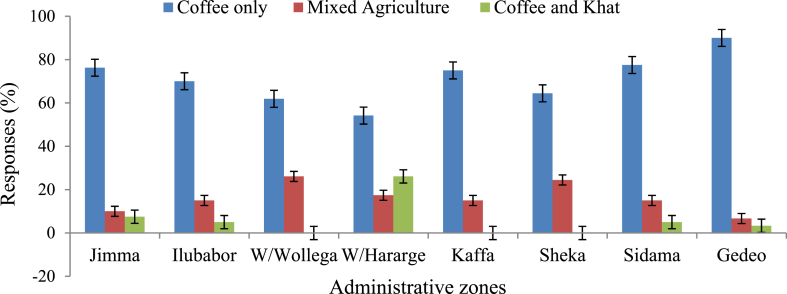
Major source of income generation of the household across major coffee-growing areas in Ethiopia.

This study confirmed that coffee was the most important cash crop being produced, with 79% of land under permanent crops and the primary source of income for 71% of the surveyed growers. In Gedeo, Sidama, Jimma, and Kaffa, most respondents (90%, 78%, 76%, and 75%, respectively) produced coffee as a primary source of cash (Fig. 2). In the West Hararghe zone, the dependence on coffee for cash was relatively low (54%) compared to the other zones. In addition to coffee, several annual and perennial crops were produced and mainly used for home consumption, with a few sold for income. The most common crops were *Persea americana, Musa acuminate, Brassica oleracea, Daucus carota, Catha edulis, Sorghum bicolor, Arachis hypogaea, Cicer arietinum, Phaseolus vulgaris, Ensete ventricosum, Zingiber officinale, Brassica oleracea, Aframomum corrorima, Zea mays, Mangifera indica, Carica papaya, Capsicum annuum, Solanum tuberosum, Ipomoea batatas, Colocasia esculenta, Eragrostis tef, Solanum lycopersicum,* and *Dioscorea* species.

The farming system is diversified because of the range of other crops produced, fitting each district's agroecological conditions. The field visit during our survey confirmed that most West Hararghe zone farmers grow coffee under an intensive mixed cropping system, mainly with *Z. mays*, *S. bicolor*, *P. vulgaris*, *A. hypogaea*, and *C. edulis*. The farmers in this zone were also known for fattening animals for income generation. Due to the alternative sources of income, mainly *C. edulis*, the production of coffee was observed to be declined. In Sidama and Gedeo zones, crops like *Z. mays*, *E. ventricosum*, *S. tuberosum*, *I. batatas*, and others are intercropped with coffee. Most of these crops were used for home consumption, and very few for income generation. Similarly, in Sheka and Kaffa Zones, coffee was intercropped with crops like *M. acuminate*, *P. americana*, *A. corrorima*, *Z. officinale*, *Piper longum*, and different vegetables that served as income generations.

#### 3.1.2 Comparison of landholdings and coffee farm size

The size of the landholding is one of the important factors in determining the economic status of farmers. The average landholding size varied across the surveyed zones ranging from as low as 0.73 ha per household in the West Hararghe zone to a high of 3.96 ha in the West Wollega zone. The prevalence of small landholdings (up to 0.5 ha) was 39% in the West Hararghe and 10% in the Gedeo zone. In contrast, in the West Wollega zone, only 2% of farmers operated small landholdings of 0.5 ha or less, while 69% had land areas greater than 2 ha. On average, 53% of the sampled households operated on land sizes of up to 2 ha, while 48% operated on land sizes greater than 2 ha. The study indicated that the mean landholding size per household was 3 ha with a standard deviation of 2. On average, 2 ha of land was allocated by farmers for coffee production. In contrast, the land allocated for other agricultural activities was 0.6 ha. This land allocation also varied from zone to zone, and the maximum (92%) land share for coffee was in the West Hararghe zone, and the minimum (71%) share was in the West Wollega zone.

#### 3.1.3 Access to extension and farm advisory service

Many respondents (73%) indicated that they have adequate access to extension services for technical support for their coffee farm. The average number of visits made by coffee experts/DAs was 1.26 times a year (SD = 1.45) and varied across locations and among the farmers. It has also been noticed that development agents and most governmental officers visited high-producing farmers up to 10 times per year. The coffee growers in the surveyed areas had access to training and technical services provided by the government and NGOs, varying from location to location. According to the survey, 68% of the respondents had received training on coffee production, but it was insufficient. The average number of training days was 3.62 (SD = 2.92), and 42% of the respondents had five to fourteen training days in the past two to three years.

### 3.2 Coffee production practices by respondents

#### 3.2.1 Coffee production systems and coffee varieties cultivated

The present study indicated that 62% of the overall respondents owned semi-forest coffee, while 29% of them owned garden coffee, and the remaining owned both semi-forest and garden coffee. The coffee production system differed among the assessed zones and from one district to another (Table S2). The production system in the West Hararghe districts was 100% garden coffee, whereas in the districts of West Wollega, Illubabor, and Kaffa, the majority of farmers produced semi-forest coffee, and farmers in the districts of Gedeo and Sidama zones produced both garden and semi-forest coffee almost equally (Table 1).

**Table 1:** Coffee production systems (CPS), variety cultivated, and age of coffee trees in 2018 cropping system for each coffee growing district, zone, and region of Ethiopia.

Variable	Region															
	Oromia									South Nations Nationality and Peoples Region						
	Jimma			Ilubabor		W/Wollega		W/Hararghe		Kaffa	Sheka		Sidama		Gedeo	
	Mana	Gomm.	LK	Yayo	Hur.	Haru	NK	Habro	DL	Gimbo	Yeki	Ander.	A .W.	W.G.	YC	Wen.
CPS																
SF	64.71^e^	63.64^g^	64.29^f^	96.43^b^	100.00^a^	100.00^a^	100.00^a^	0.00^*l*^	0.00^*l*^	95.00^c^	66.67^d^	62.50^h^	35.00^k^	40.00^i^	35.00^k^	35.29^k^
G	17.65^i^	18.18^g^	17.86^h^	0.00^j^	0.00^j^	0.00^j^	0.00^j^	100.00^a^	100.00^a^	0.00^j^	20.83^f^	25.00^e^	50.00^b^	45.00^d^	55.00^b^	47.06^c^
SF & G	17.65^a^	13.64^e^	14.29^d^	0.00^i^	0.00^i^	0.00^i^	0.00^i^	0.00^i^	0.00^i^	0.00^i^	8.33^g^	6.25^h^	15.50^b^	15.00^c^	10.00^f^	17.65^a^
Plant.	0.00^e^	4.55^b^	3.57^d^	0.00^e^	0.00^e^	0.00^e^	0.00^e^	0.00^e^	0.00^e^	0.00^e^	4.17^c^	6.25^a^	0.00^e^	0.00^e^	0.00^e^	0.00^e^
Forest	0.00^c^	0.00^c^	0.00^c^	3.57^b^	0.00^c^	0.00^c^	0.00^c^	0.00^c^	0.00^c^	5.00^a^	0.00^c^	0.00^c^	0.00^c^	0.00^c^	0.00^c^	0.00^c^
Variety																
A	64.71^e^	64.64^e^	64.29^f^	59.86^h^	62.71^g^	73.75^b^	78.88^a^	28.13^o^	31.67^n^	45.00^i^	68.00^d^	68.34^c^	39.50^k^	40.50^j^	37.10^m^	39.00^*l*^
B	4.88^p^	5.32^o^	7.14^m^	14.71^h^	13.29^i^	8.07^l^	6.13^n^	43.94^a^	42.17^b^	25.00^g^	12.25^k^	13.00^j^	35.50^d^	34.30^c^	30.05^e^	28.00^f^
A & B	30.41^b^	30.05^c^	28.57^d^	25.43^g^	24.00^j^	18.18^m^	15.00^n^	27.94^e^	26.17^f^	30.00^c^	19.75^k^	18.66^l^	25.00^i^	25.20^h^	32.85^a^	33.00^a^
Age*																
<10	9.88^g^	10.55^f^	10.71^f^	10.86^f^	8.00^i^	9.09^h^	10.00^g^	12.27^d^	11.32^e^	5.00^k^	13.17^b^	11.50^e^	7.00^j^	7.75^i^	14.26^a^	12.63^c^
11–20	11.36^m^	9.41^n^	10.71^m^	16.64^k^	19.50^i^	18.18^j^	15.00^*l*^	31.85^e^	32.80^d^	35.00^c^	24.33^h^	32.25^de^	42.00^a^	38.75^b^	28.98^g^	30.61^f^
21–30	50.87^b^	51.60^a^	50.00^c^	44.14^e^	47.00^d^	50.00^c^	50.00^c^	37.29^h^	33.52^j^	35.00^i^	41.67^f^	43.75^e^	41.00^g^	44.00^e^	50.15^c^	46.88^d^
>30	27.90^b^	28.44^a^	28.57^a^	28.36^a^	25.50^c^	22.73^e^	25.00^d^	18.59^h^	22.37^f^	25.00^d^	20.83^g^	12.50^i^	10.00^j^	9.50^k^	6.61^l^	9.87^j^

*Age of coffee plants (in year); means followed by different letter(s) within the row are significantly different (*p* < 0.05) according to Tukey's test; SF = Sefi-forest coffee; G = Garden coffee; Plant. = Plantation coffee; A = Local varieties; B = Improved varieties; W = West; LK = Limu Kosa; Hur. = Hurumu; NK = Nole Kaba; DL = Daro Lebu; Ander. = Anderacha; AW = Aleta Wendo; WG = Wondo Genet; YC = Yirgachefe; Wen. = Wenago.

Many respondents were using local varieties produced from seedlings in their nursery (55%), while improved varieties were grown by 19% of the respondents. Farmers in the West Hararghe districts grew more improved varieties than all other survey zones (Table 1). The majority of farmers in Ethiopia (65%) had very old coffee trees (more than 21 years old), and the age of coffee trees varied across the surveyed districts and zones (Table S2). Farmers in the Jimma and Ilubabor zones districts owned very old coffee trees compared to other districts. In contrast, farmers in the districts of Gedeo and Sidama owned coffee trees in productive stages (Table 1). There was a negative correlation (−0.126, p = 0.025) between coffee yield level and coffee tree age (Table S4).

#### 3.2.2 Farmers’ perception of coffee yield level

The study revealed that 43% of the respondents reported bean yield from their farm ranging from 0.31 to 0.40 t ha^−1^, but 31% of the respondents produced less than 0.30 t ha^−1^, and 24% of them obtained bean yield ranging from 0.41 to 0.50 t ha^−1^, while only 7% of them reported producing more than 0.50 t ha^−1^. Coffee yield ha^−1^ varied among the assessed zones and from one district to another (Table S3). The highest average bean yield (0.484 t ha^−1^) was recorded in the Habro and D/Lebu (0.463 t ha^−1^) districts of the West Hararghe zone followed by the districts of the Gedeo zone, whereas; the lowest coffee bean yield (0.26 t ha^−1^) was recorded from the Nole Kaba district of the West Wollega zone (Table 2). There was a negative correlation between the yield level of coffee and weed infestation level (r = −0.737, *p* < 0.0001) and positive correlations between the yield level of coffee and types of weeds (r = 0.442, *p* < 0.0001) and coffee variety cultivated (r = 0.201, *p* < 0.0001) (Table S4).

**Table 2:** Yield levels of coffee in major coffee-growing regions of Ethiopia in 2018 cropping season.

Region	Zone	District	Coffee yield levels (t ha^−1^)	
			Mean	Range
Oromia	Jimma	Mana	0.284^fg^	0.17–0.42
		Gomma	0.304^fg^	0.15–0.74
		Limu Kosa	0.325^ef^	0.15–0.79
	Illubabor	Yayu	0.390^c^	0.28–0.52
		Hurumu	0.333d^-f^	0.18–0.40
	West Wollega	Haru	0.326^ef^	0.19–0.50
		Nole Kaba	0.260^g^	0.22–0.34
	West Hararghe	Daro Lebu	0.463^ab^	0.36–0.56
		Habro	0.484^a^	0.40–0.58
SNNP*	Kaffa	Gimbo	0.387^cd^	0.28–0.53
	Sheka	Yeki	0.418^bc^	0.24–0.78
		Anderacha	0.329^ef^	0.21–0.49
	Sidama	Aleta Wendo	0.412^bc^	0.26–0.58
		Wondo Genet	0.369^c-e^	0.28–0.53
	Gedeo	Yirgachefe	0.426^bc^	0.34–0.54
		Wenago	0.399^c^	0.30–0.51

*SNNP South Nations Nationality and Peoples Region, means followed by different letter(s) are significantly different (*p* < 0.05) according to Tukey's test.

### 3.3 Coffee yield gap, farmers’ perception of crop loss and its association with weed flora

#### 3.3.1 Perception of farmers to estimate coffee yield loss due to weeds

**Fig. 3:**
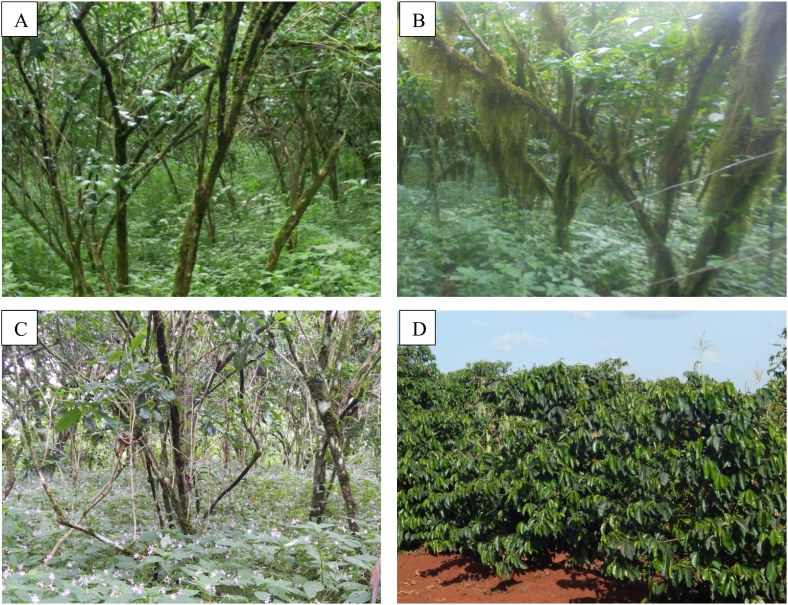
Coffee's growth performance at Jimma (A), Illubabor (B), West Wollega (C), and West Hararghe (D) zones of Ethiopia.

The investigation revealed that of the total sampled households (*N* = *320*), almost all (99.40%) (*p* < 0.001) respondents confirmed that weed is found to be a serious problem that reduces the yield and quality of the coffee (Table S3). From the field visit during the survey period, it was confirmed that coffee fields were infested with weed species (Fig. 3), and farmers claimed that weed management is one of the major and all-year-round activities that entail a high cost in coffee production. According to farmers' perception, the average yield loss of coffee due to weeds was 44%, and the estimated yield loss varied among surveyed zones and from one district to another (Table 3). Based on the farmers’ perception, the finding showed that the highest yield loss was reported from coffee farms in the Nole Kaba district (0.34 t ha^−1^), followed by the Mana district (0.337 t ha^−1^), while the minimum loss was reported in Habro and Daro Lebu (0.152 and 0.20 t ha^−1^), respectively, in the West Hararghe zone (Table 3).

**Table 3:** Estimated coffee yield loss in major coffee growing districts of Ethiopia in 2018 cropping season.

Region	Zone	District	Yield loss (t ha^−1^)	
			Mean	Range
Oromia	Jimma	Mana	0.337^a^	0.20–0.42
		Gomma	0.334^ab^	0.15–0.43
		Limu Kosa	0.313^ab^	0.10–0.49
	Illubabor	Yayu	0.284^bc^	0.13–0.49
		Hurumu	0.308^ab^	0.18–0.40
	West Wollega	Haru	0.310^ab^	0.18–0.48
		Nole Kaba	0.340^a^	0.25–0.42
	West Hararghe	Daro Lebu	0.200^f^	0.12–0.27
		Habro	0.152^g^	0.06–0.27
SNNP*	Kaffa	Gimbo	0.286^bc^	0.15–0.42
	Sheka	Yeki	0.249^c-e^	0.01–0.42
		Anderacha	0.311^ab^	0.15–0.42
	Sidama	Aleta Wendo	0.230^d-f^	0.06–0.41
		Wondo Genet	0.270^b-d^	0.11–0.36
	Gedeo	Yirgachefe	0.221^ef^	0.11–0.31
		Wenago	0.246^c-e^	0.15–0.32

*SNNP South Nations Nationality and Peoples Region, means followed by different letter(s) are significantly different (*p* < 0.05) according to Tukey's test.

The present study showed that weed infestation levels varied across the districts, zones, and regions (Table 4, S3). The coffee farms in the districts of the West Wollega and Jimma zones were highly infested with weeds. The average coffee yield gap in the survey areas, as compared to the current national average yield level (0.64 t h^−1^), was observed to be 42% and was significantly correlated (r = 0.879, *p* < 0.001) with farmers' perception of yield loss estimation mainly attributed to weed infestations in their farms in different agro-ecologies and coffee farming systems (Table S4). According to farmers’ perception assessments, more than 50% of the coffee farmers believed that the yield loss due to weeds ranged from 41% to 50%.

**Table 4:** Levels of weed infestation, weed management methods and frequency of weeding in major coffee growing regions of Ethiopia in 2018 cropping season.

Region	Zone	District	Levels of weed infestation (scale)^p^	Weed management (scale)^q^	Weeding frequency
Oromia	Jimma	Mana	2.65^a^	1.29^i^	1.35^h^
		Gomma	2.50^ab^	1.36^hi^	1.50^h^
		Limu Kosa	2.29^a-d^	1.54^f-i^	1.64^gh^
	Illubabor	Yayu	1.67^d-f^	1.93^d-g^	2.19^e-g^
		Hurumu	2.17^a-d^	1.50^g-i^	1.83^f-h^
	W/Wollega	Haru	2.32^a-c^	1.41^hi^	1.45^h^
		Nole Kaba	2.80^a^	1.20^i^	1.30^h^
	W/Hararghe	Daro Lebu	0.78^h^	2.61^ab^	3.44^ab^
		Habro	0.13^i^	2.88^a^	3.81^a^
SNNP*	Kaffa	Gimbo	1.95^b-e^	1.74^e-h^	2.21^e-g^
	Sheka	Yeki	1.67^d-f^	1.96^c-f^	2.50^de^
		Anderacha	2.44^a-c^	1.50^g-i^	1.75^gh^
	Sidama	A/Wondo	1.20^f-h^	2.20^cd^	2.90^b-d^
		W/Genet	1.85^c-e^	1.85^d-g^	2.35^d-f^
	Gedeo	Yirgachefe	0.90^gh^	2.35^bc^	3.10^bc^
		Wenago	1.41^e-g^	2.12^c-e^	2.59^c-e^

*SNNP South Nations Nationality and Peoples Region; means followed by different letter(s) within the column are significantly different (*p* < 0.05) according to Tukey's test; W = West, ^p^0 = Weeded, 1 = Slightly weeded, 2 = Slightly infested, 3 = Heavely infested; ^q^0 = No weed managemnt, 1 = Slightly weed management, 2 = Moderate weed management, 3 = Best weed management.

Weed management practices varied among zones and from one district to another (Table S3). Best weed management practices of coffee farms were observed in districts of the West Hararghe zone compared to other districts of the surveyed areas. The frequency of weeding varied among zones and from one district to another. Farmers in West Hararghe, Gedeo, and Sidama zones removed weed frequently (up to four times), with less frequently removed in districts of the West Wollega and Jimma zones. The growth performances of coffee trees from some of the study areas were varied, and at West Hararghe, the tree stands were well managed (Fig. 3D), but at Jimma (Fig. 3A), Illubabor (Fig. 3B), and West Wollega (Fig. 3C), coffee trees looked like shrubs.

#### 3.3.2 Farmers' knowledge and perceptions of the type of weeds attributed to coffee yield reduction

**Fig. 4:**
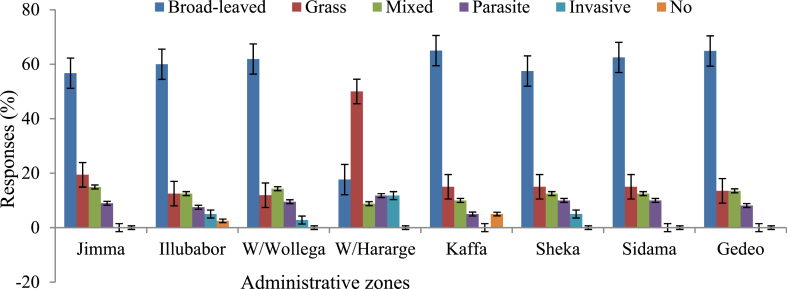
The most problematic weeds in different coffee growing zones in Ethiopia.

During our interview, farmers were asked to identify the types of weeds and list the most problematic weeds in their fields. From the total sampled households, 56% of the respondents claimed that broad-leaved weed species are responsible for coffee yield reduction, whereas; 19% indicated that grass weeds challenged them. In this survey study, it was found that there was a significant correlation (r = −0.528, *p* < 0.001) between the farmers’ knowledge of yield losses and the types of weeds in their coffee farms (Table S4). In addition to common weed species (broad-leaved and grass), the respondents also confirmed that parasitic and invasive weed species hinder their coffee yield and quality. The types and distributions of weeds varied among the zones in the type of weeds affecting coffee yield levels (Fig. 4). The coffee farms found in the South and Southwestern parts of the country are highly dominated by broad-leaved weed species, but 50% of the respondents at West Hararghe said that the grass weed species had challenged them.

According to the farmers' knowledge and perceptions, the most troublesome grass and sedge weed species were *Digitaria abyssinica, Oplismenus hirtellus, Cynodon* spp*,* and *Cyperus rotundus*. Similarly, *Achyranthes bidentata*, *Ageratum* spp*, Bidens pilosa, Commelina* spp*, Galinsoga parviflora,* and *Oxalia species* were among the most problematic broad-leaved weed species found in coffee farms. During this assessment, farmers confirmed that the presence and importance of the weed species varied from place to place. Accordingly, *Oplismenus hirtellus* and *Achyranthes bidentata* were rated as the most problematic weed species in the surveyed areas except at West Hararghe. From the farmers’ perspective, it has been noticed that there were also parasitic weed species such as *Orobanche minor*, *Cuscuta campestris*, *Tapinanthus globiferus*, *Striga hermonthica*, and ferns (such as *Cyclosorus parasiticus, Nephrolepis exaltata, Microsorum musifolium,* and *Platycerium* spp.) that affected the yield of coffee. Farmers also confirmed the existence of invasive weeds, namely *Parthenium hysterophorus* and *Mimosa pudica*, that hinder the yield of coffee. The existence and distribution of the weed species varied from zone to zone. Thus, farmers in the West Hararghe are highly challenged by grass and parasitic weed species compared to the other parts of the study areas. Information gathered during the field survey revealed that *Salvia tiliifolia, Tagetes minuta,* and *Portulaca quadfirida* were the most aggressive and categorized as weeds resistant to herbicides in the West Hararghe zone. On the other hand, *Arisaema dracontium* and *Tapinanthus globiferus* were the most problematic and difficult to control weeds, as respondents confirmed, especially in Jimma and Sheka zones because they are resistant to herbicides.

#### 3.3.3 Knowledge of farmers for weed-controlling options

Even though there are different options to manage coffee weeds, the perception assessment showed that 50% of the respondents (*p* < 0.001) used mechanical weed control methods, of which frequent slashing, hand weeding, and digging are the most commonly practiced. On the other hand, 23% of the respondents used mechanical and cultural practices to control weeds in their fields. Farmers’ weed control options differed among the assessed zones and from one district to another (Table S3). Farmers in the Jimma districts mainly used the mechanical weed control method, whereas farmers in the districts of West Hararghe used mechanical, cultural, and a combination of the two almost equally (Table 5). Farmers in the Sheka districts used herbicides to control weeds with the highest proportion as compared to the other districts, whereas no data was reported on farmers using herbicides in the districts of West Hararghe and Yirgachiefe (Table 5). All coffee plantations and a few farmers indicated they have been using herbicides to control weeds; herbicides are less labor-intensive and more efficient than mechanical and cultural weed control techniques. The respondents practiced regular slashing (every two to four weeks intervals) in their coffee farms to tackle the impact of weed infestation. However, they perceived that using this technique was often unsatisfactory as it increased the weed control cost by up to 50–70%. As a result, respondents (especially experts at large-scale private coffee farms) confirmed that the cost of weed protection had been a principal thing in their coffee production. However, if they apply herbicides (at least once), their farm is free from weeds for the cropping season. The survey assessment also revealed that large-scale private coffee farms have used cover crops such as *Desmodium* species to control weeds.

**Table 5:** Farmers' weed control mechanisms in major coffee-growing areas of Ethiopia.

Region	Zone	District	Weed control methods			
			Mechanical	Mechanical & Cultural	Herbicides	IWM
Oromia	Jimma	Mana	64.71^a^	5.88^n^	11.76^b^	17.65^i^
		Gomma	63.64^c^	9.09^m^	9.09^e^	18.18^h^
		Limu Kosa	64.29^b^	10.71^l^	10.71^d^	14.29^m^
	Illubabor	Yayu	51.85^g^	18.52^i^	11.11^c^	18.52^g^
		Hurumu	50.00^h^	16.67^k^	8.33^f^	25.00^e^
	West Wollega	Haru	45.45^k^	18.18^j^	4.55^k^	31.82^c^
		Nole Kaba	35.00^m^	25.00^f^	5.00^j^	35.00^a^
	West Hararghe	Daro Lebu	33.33^n^	33.33^d^	0.00^*l*^	33.33^b^
		Habro	31.25^o^	37.50^c^	0.00^*l*^	31.25^d^
SNNP*	Kaffa	Gimbo	52.64^f^	21.05^g^	5.26^i^	21.05^f^
	Sheka	Yeki	54.18^e^	16.67^k^	12.50^a^	16.66^k^
		Anderacha	56.25^d^	18.75^h^	12.50^a^	12.50^n^
	Sidama	Aleta Wendo	47.50^i^	32.50^e^	5.00^j^	15.00^*l*^
		Wondo Genet	42.50^l^	32.50^e^	7.50^g^	17.50^j^
	Gedeo	Yirgachefe	50.00^h^	40.00^b^	0.00^*l*^	10.00^o^
		Wenago	47.06^j^	41.18^a^	5.88^h^	5.88^p^

*SNNP: South Nations Nationality and Peoples Region; means followed by different letter(s) within the column are significantly different (*p* < 0.05) according to Tukey's test; IWM = Integrated weed management.

#### 3.3.4 Farmers' perception of applying bioherbicides/organic herbicides use in a coffee farm

During this survey assessment, it was found that 7% of farmers are using synthetic herbicides across the study areas. They all reiterated that they purchase from the local market based on the recommendation of agricultural extension services. They confirmed that they used it due to effective weed control. On the other hand, most of the respondents were using different weed control options, especially mechanical weed control rather than synthetic herbicides. According to their thoughts, even though synthetic herbicides are effective and save time and money, they have been well informed about the side effects of synthetic herbicides.

Information obtained through personal discussion with some farmers elucidated that some weed species are resistant to synthetic herbicides, and the mechanical methods (such as slashing and hand weeding) are not effective as these methods are time-consuming and laborious. The same was confirmed during FGD with farmers and expertise in the survey areas. So, it was confirmed that farmers need alternative measures to control weeds in coffee farms. Thus, it is necessary to devise methods that could reduce not only the cost of production, including saving time and labor but also with no environmental and health problems. During this survey study, the farmers were asked whether they had used bioherbicides so far or had any information about bioherbicides. The result revealed that only a few farmers and DAs knew about this weed control option. From the surveyed areas, it was also confirmed that no governmental or non-governmental organizations were working on organic herbicide activities. During the group discussion, the benefits of organic herbicides over synthetic herbicides were discussed, and it was confirmed that all respondents are willing to use bioherbicides if they are available and affordable to everyone.

#### 3.3.5 Discriminate canonical correlation analysis of coffee yield-reducing factors

The association between dependent and independent variables was determined using canonical correlation analysis. The intercorrelation matrix analysis in this study indicated a strong correlation between weed infestation and weed management (r = −0.937) and between weed infestation and frequency of weeding (r = −0.935). The weed infestation level is a very important variable describing the association with farmers' knowledge of coffee yield levels and reduction due to weeds, so weed management and frequency of weeding are excluded from the model. The predictor variables included in the model were the age of coffee trees, weed infestation, types of weeds, coffee production system, and coffee varieties cultivated. The canonical correlation analysis resulted in two canonical functions, and the first canonical correlation is 0.8923 (Table S5). This value represents the highest possible correlation between any linear combination of the yield area variables and any linear combination of the yield-limiting factor variables. The first approximate *F* value of 75.76 with the *p*-value for this function is small (*p* < 0.001); the null hypothesis would be rejected at the 0.05 level. On the other hand, the second approximate *F* value of 0.19 with the *p*-value is large (*p* = 0.9451), and it would fail to reject the null hypothesis. Hence, only the first canonical correlation was required to interpret the results.

The canonical correlation analysis resulted in an *R*_*c*_^*2*^ of 0.7962 and 0.0023 for functions 1 and 2, respectively (Table S5). Collectively, the full model across both functions was statistically significant, with a Wilks lambda (λ) of 0.2033, *F*(10, 622) = 75.76, (*p* < 0.0001) (Table S6). “Wilks λ represents the variance unexplained by the model, 1- λ yields the full model effect size in an *R*_*c*_^*2*^ metric” [26]. Across the set of two canonical functions, the *R*_*c*_^*2*^ type effect size was 0.7967, which indicates that the full model explained 79.67% of the variance shared between the two variable sets. The first function is a very high canonical correlation and highly reveals the existence of interdependence among the groups accounting for 79.62% of the variance explained, while function 2 explains a very small portion (0.23%) of the shared variance between the two variable sets.

The estimated canonical weights were used to evaluate the relative importance of variables in the model for the two sets of the two functions, as displayed in Table 6. The standardized canonical coefficients in Table 6 show that the first canonical variable for the coffee yield-related group is the weighted sum of the variables yield level (−0.193) and yield reduction (0.849). The coefficient for the yield-limiting factor variables shows that levels of weed infestation contribute heavily to the function of 1 canonical variable (0.920). This implies that a one standard deviation increase in the level of weed infestation leads to a 0.920 standard deviation increase in the score on the second canonical variate in the first variable set when the other variables in the model are held constant. Similarly, types of weeds also contribute significantly to the function 1 canonical variable (−0.081). While coffee production systems, the coffee variety cultivated and the age of coffee trees have less influence on the variables.

**Table 6:** Standardized canonical coefficients from the CANCORR procedure.

Standardized canonical coefficients for the coffee yield-related		
	Function 1	Function 2
Yield level	−0.193	1.468
Yield reduction	0.849	1.213
Standardized canonical coefficients for the yield-limiting factor		
	Function 1	Function 2
Levels of weed infestation	0.920	0.135
Types of weeds	−0.081	0.298
Coffee variety cultivated	−0.055	−0.300
Coffee production systems	−0.056	−0.213
Age of coffee trees	−0.008	−0.919

The overall relationship between variables within the sets and across both functions is presented in (Table 7). The yield-related variable, such as yield reduction, is strongly associated with the function 1 canonical variable with a correlation of 0.992. The most closely related influence is the yield level, which correlates with the canonical variable of −0.819. The correlations for the yield-limiting factor variables show that the canonical variable function 1 seems to represent all five measured variables, with the levels of weed infestation being the most influential (0.994). To some extent, the same influence is the variable types of weeds, which correlates with the canonical variable of −0.598. This measure directly identifies the relationship between the yield and yield-limiting factor variables. Hence, these results mean that coffee yield and yield-limiting factors are related. The level of weed infestation is associated with yield levels and yield reductions in coffee.

**Table 7:** Correlations between the canonical variables and the original variables.

Correlations between the coffee yield-related and their canonical variables		
	Function 1	Function 2
Yield level	−0.819	0.574
Yield reduction	0.992	0.130
Correlations between the yield-limiting factors and their canonical variables		
	Function 1	Function 2
Levels of weed infestation	0.994	−0.022
Types of weeds	−0.598	0.201
Coffee variety cultivated	−0.284	−0.242
Coffee production systems	−0.403	−0.298
Age of coffee trees	0.139	−0.879
Correlations between the coffee yield and the canonical variables of the yield-limiting factors		
	Function 1	Function 2
Yield level	−0.731	0.028
Yield reduction	0.885	0.006
Correlations between the yield-limiting factors and the canonical variables of the yield-related		
	Function 1	Function 2
Levels of weed infestation	0.887	−0.001
Types of weeds	−0.533	0.010
Coffee variety cultivated	−0.254	−0.012
Coffee production systems	−0.360	−0.015
Age of coffee trees	0.124	−0.043

## 4 Discussion

The survey revealed that most respondents used local varieties, but most farmers in West Hararghe used improved varieties. This might be because the area's environmental conditions and agricultural practices are varied from other parts of the surveyed zones. However, the other surveyed areas almost resemble environmental conditions that are suitable for the production of different coffee varieties; hence, farmers may use the varieties that are locally available and affordable. From the coffee production systems point of view, all plantation farms in the study areas have been using improved coffee varieties obtained from the Jimma Agricultural Research Center; hence they were observed to have relatively productive levels and fewer crop losses due to weeds. However, semi-forest and garden coffee production systems produced local and improved varieties with a medium yield level but increased crop losses due to weeds. That is why, during the assessment, it was observed that the yield of coffee decreased from time to time, and according to the farmers' perception, this could be due to the lack of improved varieties in the surveyed areas. The indirect analysis of factors influencing coffee yield level and yield reduction based on farmers' perceptions confirms that the cultivated coffee variety contributes to yield level and reduction. Several studies have shown the association between yield potential and cultivated variety [28,29].

The assessment showed that the majority of the respondents reported that bean yields from their farm ranged from 0.31 to 0.40 t ha^−1^, and few farmers produced more than 0.50 t ha^−1^, which was as low as compared to the national average yields (0.64 t ha^−1^) in the cropping year of 2019/20 [6]. This yield variation is due to different yield-limiting factors, mainly the level of weed infestations and types of weeds in the coffee farms, which are directly associated with the estimated yield of coffee beans in the surveyed areas. This yield variation might also be due to farmers' inability to give the correct yield estimation or the estimated national data being exaggerated because such data are mainly obtained from projections. This could also be due to farmers growing different coffee landraces with a low-yielding capacity and poor management operations. As previously reported [7], the lack of improved varieties in farmers' hands played a major role in the low yielding of coffee. It was also noticed that most respondents had a very old tree which is highly correlated with the estimated low yield of coffee beans. Hence, this could also be a reason why the yield of coffee is low in the study areas, which needs technological interventions. Teshome et al. [30] stated that the yield and quality of coffee could decrease as the plant physiologically declines. The correlation between coffee age and yield was also documented [31].

The current study also showed that the coffee yield varied across the study areas, and even if this variation might be due to agricultural operations and the environment, it was possible to detect a clear correlation between the yield gap and farmers' perceptions of yield reduction due to weeds, weed infestation and types of weeds. Tolera and Gebermedin [7] showed that farmers in the West Hararghe areas are implementing good agricultural practices with existing indigenous knowledge of weed control practices in coffee production to improve their production and productivity. Tadesse et al. [32] also reported that better support for weed management from the agricultural office, market access, and a sufficient labor force existed in the Gedeo and Sidama zones. This might be why the yield in those zones was high. Most farmers in the Jimma, Illubabor, and West Wollega zones implemented substandard agricultural practices that subjected the coffee plants to high weed competition and thus caused low coffee yields. The yield variation was also observed across coffee production systems. However, the respondents could not estimate the yield of coffee per hectare due to no or weak human intervention in the case of forest coffee production. It was noticed that the average yield increased when it moved from semi-forest to garden and from garden to plantation coffee system, which is in line with the previous report [33].

According to the current survey results, almost all respondents agreed that weeds are a serious problem that reduces the yield and quality of coffee. Abouziena and Haggag [8] also reported that weeds can reduce crop yields by more than 50%. Most respondents said broad-leaved weeds are responsible for coffee yield reduction, and even though the percentage share of grass weeds is small, farmers have been challenged by them. Thus, it is true that the yield of coffee could be decreased because of the competition for the available resources with different weed species growing in coffee fields. Teshome et al. [30] also reported the importance of these weed species groups. It was observed that the types and distributions of these weeds varied among the surveyed zones. The variation of weed growth, composition, and distribution from place to place could depend on soil and climatic factors [34]. Overall, the most troublesome grass and sedge weed species were *Digitaria abyssinica, Cynodon* spp*,* and *Cyperus rotundus*; while, *Ageratum* spp*, Bidens pilosa, Commelina* spp*, Galinsoga parviflora,* and *Oxalia species* were considered the most troublesome weeds found on coffee farms. Sahile [35] also reported the importance of those weed species in coffee fields.

The assessment showed that most respondents have been using mechanical weed control methods. However, all coffee plantations and a few farmers have been using synthetic herbicides to manage weeds with the recommendation of experts as emergency cases, even though the use of herbicides is discouraged in the country as a strategy that promotes the production of organic specialty coffee. This is because herbicides are less labor-intensive and more efficient than mechanical and cultural weed control techniques. The expense, labor-intensive, consumes a high amount of energy, and above all, results in major soil erosion problems of mechanical weed management reported [36,37]. The intensive use of herbicides to control weeds has been reported by several scholars with various drawbacks, such as disturbances to the environment and health problems for human beings [38,39]. Soti et al. [40] have shown that weed control with herbicides in conventional farming system is cost-effective but have serious health and environmental problems. However, farmers thought that the dependence on mechanical weed-controlling methods has not been adequate to control weeds as the growth of weeds is re-established after slashing and fast, especially in the Southwestern part of the country where the area is wet and humid conditions encourage the growth of weeds. The re-establishment of weeds without the need for reseeding or planning, especially during a period of high rainfall [41].

The indirect analysis of factors associated with yield levels and yield reduction of coffee was measured using canonical correlation analysis. The estimated standardized canonical coefficients, canonical loadings, and canonical cross-loadings were used to evaluate the relative importance of variables in the model for the two sets of the two functions. By interpreting the results by standardized canonical coefficients for the first function, it was found that the level of weed infestation variable has a greater contribution to increasing the variable in set 2 (dependent) and vice versa. The results show a strong relationship between the levels of weed infestation with yield levels and yield loss in coffee. Thus, this study confirms the farmers’ perception that weed infestation and types of weeds influence coffee yield potential and are also responsible for yield loss.

Even though synthetic herbicides are effective, save time, and are cost-efficient, most respondents are well-informed about the side effects of synthetic herbicides. Information obtained through personal discussions with some farmers revealed that some weed species resist synthetic herbicides, and farmers need alternatives to control weeds. In harmony with this report, several findings have been on the need for other alternative options to control weeds rather than herbicides due to the herbicidal resistance development of weeds besides the negative impact on the environment and health-hazardous ([[42], [43], [44], [45]]. Thus, it is necessary to devise methods that could reduce the cost of production, including saving time and labor and with no environmental and health problems. Except for a few farmers and DAs, most respondents need more information about bioherbicides (natural product-based herbicides). Almost all respondents were willing to use bioherbicide as long as it was available and affordable for them after clearly explaining the overview of this system.

## 5 Conclusion

Although Ethiopia is the home of *C. arabica* owing to high genetic diversity and suitable environmental and edaphic factors, the current study showed that the yield remains very low. Almost all coffee farming communities across the surveyed areas confirmed that weeds are imposing greater challenges to coffee-producing farmers as compared to diseases and insect pests across the study areas. This investigation further indicated divergent factors associated with the low yield caused by coffee yield-reducing variables in the study areas, which were examined using canonical correlation analysis. Weed infestation in association is the major yield reduction factor (82.14%), followed by types of weeds (7.23%), coffee variety cultivated (5%), coffee production systems (4.91%), and the age of coffee trees (0.71%), and the association of their interactions induced with higher levels of yield and yield losses of Arabica coffee. The current yield loss assessment, mainly attributed to weed infestations in coffee farming communities, is very high, which would be above the standard economic threshold level. Hence, farmers have been using different options to manage coffee weeds, of which the mechanical weed control method is the most commonly practiced in the study areas. Thus, it is concluded that predictor variables accounting for coffee's low yield and yield reduction need to be considered when planning future national coffee productivity enhancement strategies. In addition, this survey is a single-season study, but it indicates more survey work needs to be conducted to explore factors limiting the yield potential of coffee in its ecosystem in the country. Above all, more attention should be given to such systematic and advanced analysis of the response-predictor relationships to increase the yield levels of coffee in the country. Even though the methods used were formative and scientific, an estimation of coffee productivity levels and yield reduction mainly due to weed infestation based on farmers' knowledge and perceptions were the main limitations of this study because some farmers could not give the correct yield estimation. Besides, coffee yield-limiting factors overlap, and farmers may not identify the yield reduction due to a particular pest.

## Funding statement

This work was supported by 10.13039/501100010705Jimma University College of Agriculture and Veterinary Medicine (grant number AgVm HpSc/17/01).

## Author contribution statement

Abera Daba: Conceived and designed the experiments; Performed the experiments; Analyzed and interpreted the data; Wrote the paper.

Gezahegn Berecha and Mekuria Tadesse: Conceived and designed the experiments; Analyzed and interpreted the data; Wrote the paper.

Minilik Tsega: Contributed analysis tools or data; Wrote the paper.

## Data availability statement

Data will be made available on request.

## Additional information

No additional information is available for this paper.

## Declaration of competing interest

The authors declare that they have no known competing financial interests or personal relationships that could have appeared to influence the work reported in this paper.
