# Supplementary material for: Assessment of farmers’ knowledge and perceptions of coffee yield reduction due to weeds and their management in Ethiopia
Source: Heliyon. 2023 Aug 18;9(8):e19183. doi: 10.1016/j.heliyon.2023.e19183 (PMC10468382; doi:10.1016/j.heliyon.2023.e19183)
Supplement: Multimedia component 1 [file mmc1.docx]

**Supporting information**

**S1**: Weed survey questionnaire on assessment of coffee yield losses due to weeds and weed control options among coffee grower farmers/organizations across major coffee production regions of Ethiopia.

Dear participant, we would be grateful if you would take a moment to help us by giving us some information about coffee production in your area by answering the following questions. The research is being carried out under the terms of the Jimma University Research Review and Ethics Board.

1. **General information on the production area**
   1. Zone____________________________________________________________
   2. Woreda__________________________________________________________
   3. Kebele___________________________________________________________
   4. The production system of coffee
2. Forest
3. Semi-forest
4. Plantation
5. Garden
   1. Farmer/Organization Name___________________________________________
   2. Can you read and write? (if the interview is from farmers) 1. Yes 2. No
   3. What is the highest level of education you have reached?
   4. None
   5. Elementary
   6. Grade 7-8
   7. > Grade 9
   8. Higher education

- 1. How many people are living in your house?________
  2. Do you have an occupation other than coffee farming?

1. I do not have any
2. Civil Servant
3. Trader
4. Other (Specify)
5. **Questioners will be answered by coffee producers**
   1. Do you know the variety of coffee you are producing? Yes No
   2. If yes on question n^o^ 2.1, what type of variety you are producing?

________________________________________________

- 1. How long have you been engaged in coffee production? (in year)
  2. <6
  3. 7-10
  4. 11-14
  5. 15-18
  6. 19-22
  7. >23
  8. How you estimate the age of a coffee tree? (in year)

1. <10
2. 11-20
3. 21-30
4. >30
   1. What is the land (area) covered by coffee plants? (in hectare)_____________________
   2. What is the percentage share of coffee from the total crop you have? _______________
   3. How much would you produce? (in Kg)
   4. Per hectare_____________________
   5. Per year_______________________
5. **General information about coffee weed management**
   1. Are weeds a major problem on your coffee farm? Yes No
   2. If yes, on question n^o^ 3.1, how do you manage?

| **Methods** | **Rank** | **Frequency per year** | **Percentage share** | **Effectiveness** | **Drawback** |
| --- | --- | --- | --- | --- | --- |
| 1. **Biological** |  |  |  |  |  |
| Insects |  |  |  |  |  |
| Fungi |  |  |  |  |  |
| Bacteria |  |  |  |  |  |
| Animals (sheep, goat, etc.) |  |  |  |  |  |
| 1. **Cultural** |  |  |  |  |  |
| Crop competition |  |  |  |  |  |
| Cover crop |  |  |  |  |  |
| Intercropping |  |  |  |  |  |
| 1. **Mechanical** |  |  |  |  |  |
| Hand removal |  |  |  |  |  |
| Tillage |  |  |  |  |  |
| Mulching |  |  |  |  |  |
| 1. **Herbicide** |  |  |  |  |  |
| 1. **Others** |  |  |  |  |  |

- 1. How do you estimate the yield reduction due to weeds from the yield obtained per year? (%)

1. =<20 B. 21-30 C. 3-40 D. 41-50 E. >50
   1. List and rank the major weeds you encounter in your field:

| S.n^o^ | Scientific name | Common name | Local name | Rank |
| --- | --- | --- | --- | --- |
| 1 |  |  |  |  |
| 2 |  |  |  |  |
| 3 |  |  |  |  |

- 1. Do you use herbicides on your coffee farm?

1. Yes, regularly
2. Yes, occasionally
3. No, I do not use pesticides
   1. What initiates your use of those herbicides?
4. Own decision
5. Advice from DA
6. Advice from vendor
7. Advice from neighbors/friends
8. Others (please specify)
   1. Does your herbicide use solve your weed problem?
9. Yes
10. No
11. Do not know
    1. Does your use of herbicides increase levels of crop production?
12. Yes
13. No
14. Do not know
    1. Do you have any information about the side effect of herbicides? Yes No
    2. If yes, from where did you get it?

______________________________________________________________________

- 1. Have you had training about the use of herbicides? 1. Yes 2. No
  2. If yes, from whom?

______________________________________________________________________

***Any additional comments to be recorded relating to:***

Part 1: Background to the Household & Farm

Part 2: Coffee production

Part 3: Weed management practices

**Table S2****.** Analysis of variance Farmers' practices of coffee variety used, coffee production system, and age of coffee plants across major coffee-growing areas in Ethiopia.

| Source of variation | D.F. | Mean square | | | | | | | | | | | |
| --- | --- | --- | --- | --- | --- | --- | --- | --- | --- | --- | --- | --- | --- |
|  |  | Types of Variety produced | | | Coffee production systems | | | | | Age of coffee plants | | | |
|  |  | Local | Improved | Both | SF | G | SF & G | Plant. | Forest | <10 | 11-20 | 21-30 | >30 |
| Region | 1 | 7667.74*** | 5044.88*** | 273.52*** | 7223.15*** | 805.00*** | 2131.93*** | 31.16*** | 23.64*** | 14.75*** | 14726.56*** | 640.71*** | 8500.91*** |
| Zone (R) | 6 | 10872.83*** | 7523.11*** | 1398.76*** | 48757.31*** | 47776.87*** | 2182.82*** | 173.69*** | 85.40*** | 257.64*** | 2353.00*** | 1387.29*** | 1025.00*** |
| District (R * Z) | 8 | 62.09*** | 26.21*** | 25.84*** | 35.76*** | 21.52*** | 27.43*** | 32.04*** | 13.39*** | 18.66*** | 119.23*** | 56.39** | 126.95*** |
| Error | 304 | 0.306 | 0.240 | 0.086 | 0.012 | 0.002 | 0.021 | 0.001 | 0.001 | 1.140 | 2.607 | 2.119 | 0.469 |

*, ** and ***, significant at *p <* 0.05, 0.01 and 0.001, respectively; ns = non-significant; Both = Local and improved varieties; SF = Semi-forest; G = Garden; Plant. = plantation.

**Table S3.** Analysis of variance coffee productivity levels and yield loss with factors limiting coffee yield across major coffee-growing regions in Ethiopia.

| Source of variation | D.F. | Mean square | | | | | | | | | |
| --- | --- | --- | --- | --- | --- | --- | --- | --- | --- | --- | --- |
|  |  | Yield | Yield loss | LWI^a^ | EWMP^b^ | WF^c^ | TW^d^ | Mechanical | Mech. & cultural | Herbicide | IWM |
| Region | 1 | 0.155*** | 0.085** | 9.906*** | 4.790*** | 20.036*** | 0.933^ns^ | 552.829*** | 4664.204*** | 14.916*** | 9150.664*** |
| Zone (R) | 6 | 0.134*** | 0.103*** | 20.457*** | 8.983*** | 21.886*** | 9.882*** | 4979.653*** | 4665.067*** | 792.651*** | 2005.126*** |
| District (R * Z) | 8 | 0.024*** | 0.012** | 2.741*** | 0.876* | 1.770* | 0.579^ns^ | 198.918*** | 120.623*** | 64.787*** | 137.151*** |
| Error | 302 | 0.007 | 0.004 | 0.820 | 0.370 | 0.737 | 0.837 | 0.837 | 0.982 | 1.194 | 0.569 |

*, ** and ***, significant at *p <* 0.05, 0.01 and 0.001, respectively; ns = non-significant; ^a^LWI = Levels of weed infestation; ^b^EWMP = Extent of weed management practices; ^c^WF = Weeding frequency; ^d^TW = Types of weeds; IWM = Integrated weed management.

Table S4. Intercorrelation Matrix with descriptive statistics for the study variables in 2018 cropping season in Ethiopia.

| Variables | Yield | Yield loss | LWI^a^ | Types of weeds | Improve variety | CPS^b^ | Age of coffee tree |
| --- | --- | --- | --- | --- | --- | --- | --- |
| Yield | 1.00 | -0.737*** | -0.727*** | 0.442*** | 0.201*** | 0.286*** | -0.126** |
| Yield loss |  | 1.00 | 0.879*** | -0.528*** | -0.253*** | -0.382*** | 0.117* |
| LWI |  |  | 1.00 | -0.544*** | -0.217*** | -0.345*** | 0.146** |
| Types of weeds |  |  |  | 1.00 | 0.154** | 0.154*** | -0.060^NS^ |
| Improve variety |  |  |  |  | 1.00 | 0.315*** | -0.119* |
| CPS |  |  |  |  |  | 1.00 | 0.012^NS^ |
| Age of coffee tree |  |  |  |  |  |  | 1.00 |

*, **, *** significant at *p* < 0.05, 0.01, and 0.001, respectively, *N*.S. Non-significant, ^a^LWI Level of weed infestation, ^b^CPS Coffee production systems.

**Table S5.** Canonical correlations, eigenvalues, and likelihood tests.

| Function | Canco. | Adjusted  Canco | Approx  S.E. | Squared  Canco | Eigenvalues of Inv(E)*H  = CanRsq/(1-CanRsq) | | | Test of H0: The canonical correlations in the current row and all that follow are zero | | | | |
| --- | --- | --- | --- | --- | --- | --- | --- | --- | --- | --- | --- | --- |
|  |  |  |  |  | Eigenvalue | Proportion | Cumulative | LHR | Approx  F Value | Num DF | Den DF | Pr > F |
| 1 | 0.8923 | 0.8908 | 0.0114 | 0.7962 | 3.9077 | 0.9994 | 0.9994 | 0.2033 | 75.76 | 10 | 622 | <.0001 |
| 2 | 0.0489 | -0.0475 | 0.0560 | 0.0024 | 0.0024 | 0.0006 | 1.0000 | 0.9976 | 0.19 | 4 | 312 | 0.9451 |

Canco = Canonical correlation; Approx = Approximate; S.E. = Standard error; LHR = Likelihood Ratio; Multivariate tests of significance (S = 2, M = 1, N = 154.50).

**Table S6.** Multivariate statistics and approximate *F* tests.

| Statistic | Value | F Value | Num DF | Den DF | Pr > F |
| --- | --- | --- | --- | --- | --- |
| Wilks' Lambda | 0.20327588 | 75.76 | 10 | 622 | <.0001 |
| Pillai's Trace | 0.79862895 | 41.48 | 10 | 624 | <.0001 |
| Hotelling-Lawley Trace | 3.91005199 | 121.34 | 10 | 463.76 | <.0001 |
| Roy's Greatest Root | 3.90765396 | 243.84 | 5 | 312 | <.0001 |

*Note*: *F* statistic for Roy’s Greatest Root is an upper bound.
